# Phosphoethanolamine and the danger of unproven drugs

**DOI:** 10.3332/ecancer.2016.681

**Published:** 2016-10-17

**Authors:** Noam Pondé, Evandro de Azambuja, Felipe Ades

**Affiliations:** 1 Institut Jules Bordet, Brussels 1000, Belgium; 2 Centro de Oncologia e Hematologia, Hospital Israelita Albert Einstein, São Paulo 05652-900, Brazil

**Keywords:** phosphoethanolamine, cancer, science, Brazil, ethics

## Abstract

The use of unproven forms of therapy in cancer treatment is very common. In Brazil, the distribution by researchers to patients of an investigational agent called phophoethanolamine (PHOS) has led to a widely publicized scientific scandal. PHOS is a precursor to components of the cell membrane, with some published pre-clinical studies suggesting cytotoxic activity in cancer cells. The willingness of courts and of legislators to guarantee access to PHOS in spite of the lack of any clinical data and against the recommendations of scientific and medical organisations underscores the risks that unproven agents pose to regulatory authorities, health care systems and patients, and bears resemblance to other cases such as the controversy surrounding the approval of zidovudine for AIDS treatment by the FDA.

## Introduction

Brazil is a developing nation where a significant portion of the population lacks access to health care [1]. Like other developing countries, Brazil has struggled since the 1980s to build its public health care system – the Sistema Único de Saúde – and has been able to develop effective policies in certain fields of public health, such as its HIV/AIDS programme [2]. In spite of this progress, in oncology, many patients still lack access to appropriate screening and treatment [3]. In a country where daily clinical practice is suboptimal, developing clinical research poses an even bigger challenge [4], with problems ranging from lack of support and funding from health authorities [5], to very complex regulations by different regulatory authorities [6]. Investigator initiated studies remain rare, though participation in international pharma-sponsored trials has risen in recent years [5, 7]. In this setting, the full development of a Brazilian anticancer drug – from laboratory to clinical use – would be an unusual success story.

An ongoing research scandal regarding an experimental substance demonstrates how the lack of experience with the principles of clinical research among some physicians, legal entities, the media and patients can be dangerous to a countries´ health care system and exposes patients to unknown harms. Its name is phosphoethanolamine, and it bears close resemblance to other, international research-related controversies.

## Phosphoethanolamine

In August 2015, the Brazilian media began reporting on the arrest of a former salesman who was manufacturing and distributing phosphoethanolamine (PHOS) [8]. He obtained it from a retired professor at the University of Sao Paulo and was later taught how to produce it [8]. According to Brazilian law, it is a crime to distribute substances with medicinal purposes without the substance being registered by ANVISA (the Brazilian vigilance agency, equivalent to the FDA) [9]. The aforementioned professor, according to his own words [10], had been conducting research on PHOS for over 20 years. He claimed to have taken part in clinical trials at a reputable cancer centre, although the centre has denied it [11]. No clinical data on this substance have ever been published. Following termination of the alleged trials, he decided to keep distributing PHOS to patients. Word of mouth and tales of ‘miraculous’ recoveries steadily increased the number of patients that looked for PHOS [12]. The growing number of people coming to the university eventually attracted the attention of university authorities, leading them to stop this unlawful distribution in June 2014 [13].

PHOS is a precursor to phosphatidylcholine and phosphatidylethanolamine, both components of the cell membrane. Preclinical results suggest that PHOS is capable of supressing tumour growth both *in vitro and in vivo* (mouse models) though the exact mechanism has not yet been elucidated [14–15]. No data on the potential mutagenicity nor carcinogenicity is available, nor other preclinical data needed to proceed to *in human* studies, as first- and second-generation reproduction toxicity and toxicokinetics [16]. At this point, according to published data, PHOS is comparable with hundreds of other substances that have demonstrated pre-clinical antitumor activity. Unfortunately, only 5% of cancer drugs that succeed preclinically will reach the clinic [17].

After the interruption of distribution by USP, many patients went to court demanding continued supply of PHOS [18]. This judicial imbroglio with courts taking turns either authorising or stopping distribution, led to increasing pressure by patients on legislators to act. In April 2016, both houses of the Brazilian congress approved a law authorising the use of PHOS in a landslide vote [19–20]. This law has been subsequently annulled by the Supreme Court in May 2016 [21]. In parallel, phase-1 studies were started in public hospitals and are ongoing [22], despite the lack of activity in recent (unpublished) preclinical studies developed by a task force designated by the Science and Technology Ministry [23]. ANVISA, the world health organisation (WHO), Brazilian medical associations [24–26], clinical oncology researchers and well-known medical oncologists [27] have stated the experimental nature of PHOS, and the inappropriateness of its clinical use at this moment, as well as the threat posed by legislative action on this matter.

## Ethical Issues Involved

The use of unproven or complementary treatments is not new in oncology. Studies estimate that up to 70% of patients have used some kind of alternative therapy and almost all have heard about them [28]. As they are perceived by the layperson as ‘natural’ or ‘harmless’, many patients find them preferable to the rigours of ‘orthodox’ cancer treatments. This practice is risky as it can interfere with and hamper the effects of conventional treatment as, for example, with St. John’s wort and green tea [29]. Others can have important side effects and increase those related to chemotherapy, besides contamination and infection issues. An even more serious problem is the abandonment of proven treatments to follow therapies with unknown effects against cancer, if they have any effect at all, as in some of the reported PHOS cases [12, 30].

No drug candidate should be used in daily practice without properly conducted clinical research. The criteria to admit a drug into the armamentarium of cancer therapy are stringent and exist, ultimately, to serve patients. Clinical research is regulated by a set of ethical principles that have their origins in the Nuremberg trials of Nazi crimes against humanity [31]. These principles are compiled in the Declaration of Helsinki [32], which states that ‘It is the duty of the physician to promote and safeguard the health, well-being and rights of patients, including those who are involved in medical research […]. Medical research is subject to ethical standards that promote and ensure respect for all human subjects and protect their health and rights’. All clinical research must be reviewed and approved by ethics committees/institutional review boards (EC/IRB) [33], which exist to protect research subjects. Informed consent [34] must be obtained from all trial subjects and are based on accurate data about the potential benefits of an experimental agent. Exposing a patient to a compound outside of this framework is considered a serious ethical infringement against the patient’s dignity and wellbeing. The results of research conducted outside this framework are inadmissible as the basis of medical decision-making.

The scientific British Royal Society motto is *Nullius in verba,* that is, ‘on no one’s word’. It underscores the principle that science is based on data, ‘to verify all statements by an appeal to facts determined by experiment’ [35]. One of the most surprising aspects of the PHOS story was the magnitude it reached as well as the fast dissemination of this alleged ‘cancer cure’. Patients coming to their doctors with information acquired from non-scientific sources – other patients, friends, books and especially the Internet is not unusual. In this particular case, social media has been used not only by patients looking for the substance but also by those responsible for its distribution as a tool to disseminate inaccurate information and to organize political action. The mixture of a drug developed by Brazilian researchers (nationalism), questionable theories about why the scientific community rejected the use of PHOS and the dissemination in both regular and social media of interviews with alleged cured patients was the fuel for lay people to spread the news rapidly.

PHOS is one local example of a much wider issue – namely the problematic relationship between regulatory agencies, pharmaceutical companies, researchers, patients (and their support groups), and media. An acrimonious debate in late 1989 surrounded the speeded up approval by the FDA of zidovudine (AZT) as the first treatment for AIDS based on one small phase-2 trial with short follow-up [36]. The quality of trial conduction including possible breaches of blinding due to the pronounced toxicity of AZT, as well as other issues, led to the questioning of its results. At the same time, the societal impact of the AIDS pandemic led to protests both against AZT having been approved in the first place and to AZT being distributed to all patients for free [37–38]. The AZT example underscores not only the importance of strict decision making regulatory authorities but also how dangerous responding to public outcry can be.

## Judicialisation

Judicialisation is defined as the interference of the judiciary into the affairs of the executive or legislative bodies [39]. In Brazil, as in other countries [40], the judicialisation of health care is controversial. The frequent use of the Justice system to force payment of drugs and procedures not registered by regulatory health authorities is a growing issue in public health [40] and puts into question the very right of the state to regulate in this area. In Brazil, the courts may grant to claimants access to treatments or procedures not usually available in public hospitals or not yet authorised by ANVISA, but authorised by the FDA or equivalent regulatory authorities in other countries. These decisions are commonly based on the ‘right to health’ clause of the Brazilian constitution [41] that defines health as a basic human right that must be guaranteed by the state. The case of PHOS is a particularly alarming development, with courts approving the use of a substance that has never been properly tested in humans – and that thus cannot be said to promote or preserve health – a dangerous precedent for judicial demand of any form of non-proven treatment. The decision by the legislative branch to approve PHOS has also challenged the authority of ANVISA and set a worrisome precedent.

## Conclusion

In conclusion, the manufacture and distribution of unproven drugs to patients outside the framework of a clinical trial, with proper study design, approval by IRB/EC and with informed consent, infringes the ethical principles of clinical research and puts the health of patients at risk. Furthermore, approval of drugs based on substandard evidence can also lead to risks to patients and to the regulatory process itself. Thus, regulatory agencies must make decisions disregarding any existing social pressures. Judicialisation, in the case of PHOS, questions the role of government regulation of medicinal substances and of what can or cannot be defined as health care. Health decisions must be made based on scientific evidence and not on beliefs and opinions. *Nullius in Verba.*

## List of abbreviations

PHOS: Phosphoethanolamine
FDA: Food and Drug Administration
ANVISA: Agência Nacional de Vigilância Sanitária
AZT: Zidovudine
AIDS: Acquired Immune Deficiency Syndrome

## Conflicts of interest

All three authors are equally responsible for design, writing, review, and approval of the final paper. None of the authors have conflicts of interest regarding the subject of this paper.
